# Systematic Identification of the Functional lncRNAs During H7N9 Avian Influenza Virus Infection in Mice

**DOI:** 10.3390/v18030353

**Published:** 2026-03-13

**Authors:** Guoqing Wang, Zenglei Hu, Xinxin Cai, Shunlin Hu, Min Gu, Xiaoquan Wang, Daxin Peng, Jiao Hu, Xiufan Liu

**Affiliations:** 1 Key Laboratory of Avian Bioproducts Development, Ministry of Agriculture and Rural Affairs, College of Veterinary Medicine, Yangzhou University, Yangzhou 225009, China; 1986guoqing@163.com (G.W.); zengleihu@163.com (Z.H.); 19850508323@163.com (X.C.); slhu@yzu.edu.cn (S.H.); gumin@yzu.edu.cn (M.G.); wxq@yzu.edu.cn (X.W.); pengdx@yzu.edu.cn (D.P.); 2 Jiangsu Co-Innovation Center for Prevention and Control of Important Animal Infectious Diseases and Zoonosis, College of Veterinary Medicine, Yangzhou University, Yangzhou 225009, China; 3 Key Laboratory of Prevention and Control of Biological Hazard Factors (Animal Origin) for Agri-Food Safety and Quality, Ministry of Agriculture of China (26116120), College of Veterinary Medicine, Yangzhou University, Yangzhou 225009, China; 4 Joint International Research Laboratory of Agriculture and Agi-Product Safety, Ministry of Education of China, Yangzhou University, Yangzhou 225009, China

**Keywords:** H7N9 influenza virus, mice, high-throughput sequencing, lncRNA, antiviral role

## Abstract

Accumulating studies have identified the pivotal role of long non-coding RNAs (lncRNAs) in participating in host–virus interactions during virus infections. However, the regulatory roles of lncRNAs in influenza A virus (IAV) infection are still not fully elucidated. In this study, using high-throughput sequencing, we comprehensively compared the expression profiles of lncRNAs and mRNAs in mouse lungs infected either with the nonpathogenic parental (SDL124) H7N9 virus or its moderately pathogenic mouse-adapted (S8) variant. A total of 7636 significantly differentially expressed (SDE) lncRNAs were obtained in the S8-infected group compared to the mock group. As for the SDL124 group, 1042 SDE lncRNAs were identified. Subsequently, the mRNAs co-expressed with SDE lncRNAs were subjected to functional annotation and pathway enrichment analysis. The results indicated that the target mRNAs regulated by the S8 virus were mainly enriched in various immunological processes and exhibited a strong correlation with inflammatory-related signaling pathways. Moreover, 12 lncRNAs and 10 mRNAs co-expressed with SDE lncRNAs were selected and successfully verified by RT-qPCR. Among these lncRNAs, NONMMUG032982.2 and NONMMUG032328.2 exhibited strong antiviral activity against IAV. Additionally, these two lncRNAs were chosen for further in-depth bioinformatics analysis, including transcription factor prediction, coding capacity assessment, genomic location, construction of secondary structure, and prediction of potential interacting proteins. Taken together, these findings provide a cluster of lncRNAs probably associated with the virulence of IAV in mice and shed light on the anti-IAV effects of two functional lncRNAs, establishing a molecular foundation for further exploring the regulatory mechanisms of lncRNAs in IAV infection.

## 1. Introduction

Influenza A virus (IAV) is a single-stranded, negative-sense, enveloped RNA virus that belongs to the family *Orthomyxoviridae*, whose genome consists of eight RNA segments encoding more than ten viral proteins [1,2]. Owing to their cross-species transmission ability, IAVs (especially the H7N9 and H5N1 subtypes) can cause severe human infections and result in a high mortality rate [3,4]. The first human case of H7N9 avian influenza virus (AIV) infection emerged in early 2013 in China. Thus far, H7N9 AIVs have caused 1568 human cases with a mortality rate of nearly 40% (https://www.who.int/publications/m/item/influenza-update-n--554, accessed on 26 November 2025). In the past decade, H7N9 viruses have continued to pose challenges to the poultry industry and public health given their pandemic potential and virulence to humans [5]. To combat the threat of influenza viruses, vaccines and antiviral drugs are currently being used and are still under development. However, due to frequent mutations of the viruses, apart from the measures stated above, novel therapies targeting host factors that modulate AIV replication are still warranted for further research.

In recent years, long non-coding RNAs (lncRNAs) have attracted extensive attention due to their versatile roles in regulating host–pathogen interactions. LncRNAs are defined as transcripts longer than 200 nucleotides, with poor protein-coding capacity and extensive expression in mammalian cells [6,7,8]. Conventional studies on host transcriptional responses to viral infection mainly focus on protein-coding genes. However, more than 98% of the transcripts in mammals are non-coding RNAs, the majority of which are lncRNAs [9]. Most lncRNAs are transcribed by RNA polymerase II, undergo splicing, possess a cap at their 5′-end, and receive a 3′-end polyadenylated tail [10,11,12,13]. LncRNAs have a relatively low expression level, whose expression is cell type- or tissue-specific and varies at different developmental stages [14]. In addition to host-derived lncRNAs, some viruses can also generate lncRNAs [15]. Due to the low binding fidelity of RNA polymerase and poor sequence conservation across species [16], lncRNAs were previously deemed transcriptional junk [9,14]. However, numerous studies have revealed that lncRNAs play substantial regulatory roles in various biological processes, including immune response, viral replication, cell differentiation, cell cycle control, and apoptosis [17,18,19,20]. For instance, lncRNA *ALPHA* (antiviral lncRNA prohibiting human alphaviruses) interacts with CHIKV (chikungunya virus) genomic RNA to inhibit its replication [21]. Furthermore, lncRNAs modulate gene expression at the epigenetic, transcriptional and post-transcriptional levels by inducing chromatin remodeling, regulating transcription factor activities, modulating alternative splicing patterns, regulating protein activities, altering protein localization, and sponging miRNAs [1,6,22,23,24,25,26].

Currently, the diverse functions of lncRNAs during viral infection have been gradually unveiled. For example, LncRNAs can exert an antiviral immune response to impair IAV replication, such as lnc-RPS6P3, AVAN, GBP1P1 (guanylate-binding protein 1 pseudogene 1), IVRPIE (inhibiting IAV replication by promoting IFN and ISGs expression), lnc-ISG20, lncRNA-155, and *USP30-AS1* [1,2,24,27,28,29,30]. AVAN binds directly to TRIM25 and enhances K63-linked ubiquitination of RIG-I to promote an anti-IAV immune response [27]. However, in turn, viruses are also capable of hijacking host lncRNAs to antagonize antiviral effects for their own replication [6,12,26,31,32,33]. For IAV, lncRNA IPAN (influenza virus PB1-associated non-coding RNA) can stabilize viral RNA polymerase PB1 and prevent its degradation to facilitate IAV replication [6]. Moreover, lncRNAs can also play a dual role in viral infection to maintain immune homeostasis [34]. For instance, DFRV (dual function regulating influenza virus) functions as a dual-regulator, generating two distinct transcript variants during IAV infection. DFRV-long suppresses IAV replication, whereas DFRV-short exerts the opposite effect [34]. In a prior study, we identified two H5N1-inducible lncRNAs, designated Lnc45 and LncRNA#61 [35,36], which exhibited anti-IAV activity by suppressing viral polymerase activity and impeding nuclear aggregation of viral proteins. Considering the remarkable roles of lncRNAs and the significant threat of IAVs to global health, it is necessary to further elucidate the regulatory roles and underlying mechanisms of lncRNAs in response to IAV infection.

We previously characterized a pair of H7N9 viruses, SDL124 and S8, with similar genetic background but markedly different virulence in mice [3]. The S8 variant, derived from the wild-type SDL124 strain through serial murine passage, exhibits moderate pathogenicity in mice, whereas the parental SDL124 virus is avirulent for mice. Herein, using high-throughput sequencing, we comprehensively compared the expression profiles of lncRNAs and mRNAs in mouse lungs infected either with SDL124 or the S8 virus. Then, significantly differentially expressed (SDE) lncRNAs between S8 or SDL124 and the mock groups were further identified. Meanwhile, we performed functional annotation and pathway enrichment analysis of mRNAs co-expressed with SDE lncRNAs according to the GO and KEGG databases. Subsequently, we initially evaluated the potential functions of two lncRNAs in AIV infection and analyzed their relevant bioinformatic characteristics. Collectively, our results provide a catalog of lncRNAs differentially regulated by these two viruses, highlighting the important implications of lncRNAs in regulating AIV replication as well as laying the basis for further studies on the biological function of lncRNAs during AIV infections.

## 2. Materials and Methods

### 2.1. Cells, Viruses and Animals

NCTC clone 929 (L929) cells (ATCC^®^ CCL-1, Manassas, VA, USA) were cultured in Dulbecco’s Modified Eagle’s Medium (DMEM; Gibco, Waltham, MA, USA) supplemented with 10% fetal bovine serum (FBS; Gibco, Waltham, MA, USA) and maintained at 37 °C with 5% CO_2_. The H7N9 influenza viruses, A/chicken/shandong/SDL124/2015 (SDL124) and A/mouse/shandong/S8/2015 (S8) [3] were propagated in 10-day-old specific-pathogen-free (SPF) embryonated chicken eggs. Six-week-old female BALB/c mice (Yangzhou University Experimental Animal Center) were used for RNA sequencing and in vivo evaluation of viral replication, lncRNA expression, and cytokine production level. Data collection for this study took place from October 2022 to March 2024.

### 2.2. Mouse Experiments

To harvest mouse lungs for RNA sequencing, a total of 9 healthy six-week-old female BALB/c mice (Yangzhou University Experimental Animal Center, randomly divided into 3 groups, *n* = 3 per group) were anesthetized via intraperitoneal (IP) injection of Telazol^®^ (Zoetis; 55–60 mg/kg), and subsequently inoculated intranasally with 10^6^ EID_50_ in 50 μL PBS of S8 or SDL124 virus. Mice inoculated with 50 μL of PBS were served as the mock group. Three mice in each group were euthanized via IP injection of sodium pentobarbital (Zoetis; 150–200 mg/kg) at 2 days post infection (dpi), and lung samples were collected quickly and frozen immediately in liquid nitrogen until RNA extraction.

A total of 42 healthy six-week-old female BALB/c mice (Yangzhou University Experimental Animal Center, randomly grouped into 3 groups, *n* = 14 per group) were anesthetized via intraperitoneal (IP) injection of Telazol^®^ (Zoetis; 55–60 mg/kg) and inoculated intranasally with 10^5^ EID_50_ in 50 μL PBS of S8 or SDL124 virus. The mock control mice were inoculated with 50 μL of PBS. To minimize potential confounders, the experiment results were recorded by different observers who were blinded to the animal group allocation. Three mice from each group were euthanized via IP injection of sodium pentobarbital (Zoetis; 150–200 mg/kg) at 1, 3 and 5 dpi and their lungs were harvested for virus titration, cytokine detection, histopathological examination and lncRNA quantification. Data were represented as the mean ± standard deviation (SD). The remaining five mice from each group were monitored for 14 days for mortality, morbidity and body weight changes. Mice that lost 25% of their initial weight were regarded as dead and euthanized [3].

### 2.3. RNA Isolation, Library Preparation and Illumina Sequencing

Total RNAs from mouse lung samples were extracted using TRIzol^TM^ reagent (Invitrogen, Waltham, MA, USA) according to the manufacturer’s instructions. The concentration and RNA integrity number (RIN) of the purified RNAs were assessed using Agilent 2100 Bioanalyzer (Agilent RNA 6000 Nano Kit; Agilent Technologies, Santa Clara, CA, USA), and the purity of total RNAs was detected by NanoDrop^TM^. RNA samples with RIN greater than 9.0 were retained for library preparation and RNA sequencing. For the preparation of sequencing libraries, in brief, total RNA samples were treated with Ribo-Zero^TM^ Magnetic Kit (Epicentre Biotechnologies, San Diego, CA, USA) to remove ribosomal RNA (rRNA), and then the rRNA-depleted RNAs were fragmented into small pieces using fragmentation reagent. Next, the first-strand cDNA was synthesized using random primers, followed by a second-strand cDNA synthesis. The double-stranded cDNA fragments subsequently underwent end-repair and 3′-terminal adenylation followed by adapter ligation. Thereafter, adapter-ligated cDNA samples were amplified by PCR, and the products were purified with AMPure XP Beads (Beckman Coulter, Brea, CA, USA). The quality of the libraries was assessed using Agilent 2100 Bioanalyzer. Finally, qualified libraries underwent cluster generation and were sequenced on the Illumina HiSeq X Ten with 100 bp paired-end reads (PE100) generation.

### 2.4. Mapping, Transcriptome Assembly and LncRNA Identification

Following the screening strategy for mouse lncRNAs, clean reads were obtained by removing reads containing rRNA sequences, poly-N, adapter sequences, and low-quality reads from the raw reads. All high-quality paired-end clean reads were used for downstream analyses. Firstly, clean reads were aligned to the mouse mm10 reference genome using HISAT2 v2.2.0. Subsequently, aligned reads were assembled into transcripts using StringTie v2.1.5. Uniquely mapped reads were used to calculate the read number and FPKM value for each gene. Lastly, based on stringent screening steps to filter lncRNAs, the candidate set comprising both novel and known lncRNAs was obtained from the assembled transcripts.

### 2.5. Identification of Significantly Differentially Expressed Genes

Gene expression levels were quantified using FPKM values, which were calculated through StringTie v2.1.5 to normalize transcriptional abundance. Differential expression analysis for lncRNAs and mRNAs between the infected and the mock groups was subsequently performed using the edgeR package. The *p* value and false discovery rate (FDR) for each gene were determined based on the negative binomial distribution model. Finally, genes with |log_2_ fold change| ≥ 1, a *p* value < 0.05 and an adjusted *p* value (FDR) < 0.05 were classified as SDE genes between S8 or SDL124 and the mock groups.

### 2.6. LncRNA/mRNA Co-Expression Network Construction and Functional Analysis of SDE LncRNAs

LncRNAs regulate target genes in a cis- or trans-acting manner. So, we first predicted the target mRNAs of SDE lncRNAs based on cis/trans-regulatory algorithms. The mRNAs located within a window of 10 kbp upstream or downstream of the SDE lncRNAs were defined as cis-acting target genes. If the distance exceeded the threshold, we employed RNAplex software to calculate the lncRNA-mRNA binding energy. The mRNAs with the binding energy < −30 were considered as trans-acting targets. Then, we calculated the Pearson correlation coefficient (*r*) to analyze the correlation between SDE lncRNAs and target mRNAs. For the S8 group, |*r*| > 0.999 and FDR < 0.05 were used as thresholds to screen co-expressed lncRNA-mRNA pairs, while the SDL124 group applied |*r*| > 0.98 and FDR < 0.05 as screening criteria. The lncRNA-mRNA co-expression network was ultimately constructed and exported using Cytoscape v3.8.2.

To explore the functions of SDE lncRNAs, we performed enrichment analysis of the target mRNAs predicted above. GO functional annotation was performed using g:Profiler (https://biit.cs.ut.ee/gprofiler, accessed on 10 August 2025), and KEGG pathway enrichment analysis was implemented using KOBAS 3.0 [37]. GO terms and pathways with *p* value < 0.05 were considered significantly enriched.

### 2.7. Reverse Transcription Quantitative Real-Time PCR

To verify the expression of lncRNAs, host mRNAs and viral NP mRNA, total RNAs from mouse lungs and cells were extracted using TRIzol^TM^ reagent. The cDNA was synthesized using PrimeScript^TM^ RT reagent Kit (Takara, Shiga, Japan), and reverse transcription quantitative real-time PCR (RT-qPCR) was performed on a LightCycler 480 System using TB Green^®^ *Premix Ex Taq*^TM^ II (Takara, Shiga, Japan). The β-actin gene was used as an internal control. Relative expression of candidate genes was calculated using the 2^−ΔΔ^*^CT^* method. All the experiments were performed in triplicate. Data are represented as mean ± SD from three independent experiments. Primer sequences used in this study are listed in Table S1.

### 2.8. Plasmids and Viral Infection

The full-length sequences of mouse NONMMUG032982.2 and NONMMUG032328.2 were synthesized by GenePharma Company (Suzhou, China) and cloned into the empty vector pcDNA3.1(+) (EV) to yield the lncRNA expression constructs. The fidelity of these two plasmids was confirmed by DNA sequencing. To evaluate the effects of these two lncRNAs on viral replication, L929 cells were transfected with these two lncRNA plasmids or EV. At 24 h post transfection (hpt), cells were infected with the S8 virus at a multiplicity of infection (MOI) of 0.01. The expression of lncRNAs and viral NP mRNA in cells at 24 and 36 h post infection (hpi) was quantified by RT-qPCR.

### 2.9. Bioinformatics Analysis of Functional LncRNAs

The potential transcription factors (TFs) of candidate lncRNAs were predicted by combining the UCSC Genome Browser (https://genome.ucsc.edu/, accessed on 10 August 2025) and the JASPAR database (https://jaspar.elixir.no/, accessed on 10 August 2025). The genomic location of candidate lncRNAs was annotated through the UCSC Genome Browser. The coding potential of the lncRNAs was assessed by PhyloCSF tracks (https://data.broadinstitute.org/compbio1/PhyloCSFtracks/trackHub/hub.txt, accessed on 10 August 2025). The secondary structure analysis of lncRNAs was performed through the RNAfold web server (http://rna.tbi.univie.ac.at/cgi-bin/RNAWebSuite/RNAfold.cgi, accessed on 10 August 2025). Moreover, potential interacting proteins for the lncRNAs were computationally predicted using the online tool catRAPID (https://service.tartaglialab.com/page/catrapid_omics_group, accessed on 10 August 2025).

### 2.10. Statistical Analysis

All data were analyzed based on Statistical Product and Service Solutions (SPSS) statistics software (IBM Corp., Armonk, NY, USA; version 23.0). Comparisons between groups were performed using the Independent Samples *t*-test. * (*p* < 0.05), ** and ## (*p* < 0.01), *** and ### (*p* < 0.001), **** and #### (*p* < 0.0001) are considered statistically significant, and ns indicates non-significance.

## 3. Results

### 3.1. Pathogenicity Assessment of S8 and SDL124 Virus in Mice

**Figure 1:**
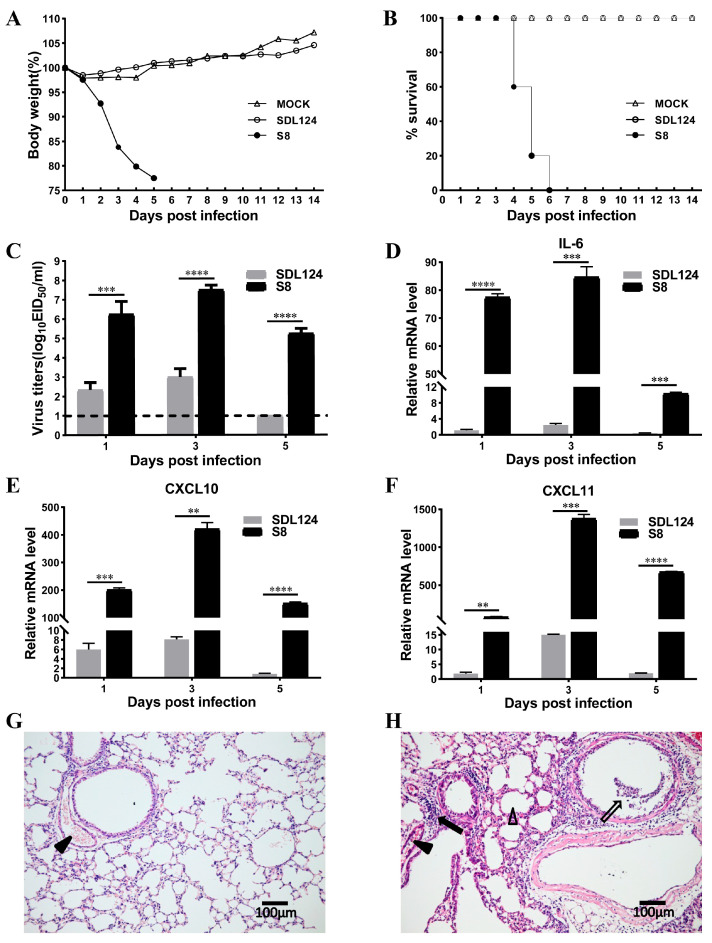
Pathogenicity assessment of S8 and SDL124 in mice. (**A**,**B**) Six-week-old female BALB/c mice (*n* = 5 per group) were infected intranasally with 10^5^ EID_50_ in 50 μL PBS of S8 or SDL124 virus, and mice inoculated with 50 μL of PBS served as the mock group. (**A**) The average weight change in mice infected with S8 or SDL124 was shown. (**B**) Survival rate of mice infected with the indicated viruses was shown. (**C**–**F**) Mice (*n* = 9 per group) were inoculated with 10^5^ EID_50_ in 50 μL PBS of each virus, and then were euthanized at 1, 3 and 5 dpi to collect lungs for virus titration (**C**) and cytokine detection by RT-qPCR (**D**–**F**). (**G**,**H**) Histopathological changes in SDL124 (**G**) and S8 (**H**) virus-infected mouse lungs at 3 dpi (scale bar, 100 μm). Black arrow: lymphocyte infiltration around blood vessels and bronchi. Empty arrow: infiltration of lymphocytes, inflammatory cells and epithelial cells shedding from alveoli, alveolar sacs and alveolar ducts. Black triangle: hemorrhages. Empty triangle: mild alveolar distension. Data shown are the mean ± SD of three independent experiments. The detection limit (the dotted line) for virus titers is 1.0 log_10_ EID_50_/_mL_. Comparisons between groups are performed using the Independent Samples *t*-test. ** (*p* < 0.01), *** (*p* < 0.001), and **** (*p* < 0.0001) are considered statistically significant.

We first systematically compared the pathogenicity of these two viruses. At a dose of 10^5^ EID_50_, all mice infected with the parental SDL124 virus survived during 14 days of observation and exhibited only mild body weight loss (Figure 1A,B). In contrast, mice inoculated with the mouse-adapted S8 variant displayed severe weight loss and succumbed to infection by 6 dpi (Figure 1A,B). Additionally, the S8 virus replicated to significantly higher viral titers in mouse lungs than the SDL124 strain at each time point of infection (Figure 1C). Given the important role of *CXCL11*, *CXCL10*, and *IL-6* in inflammatory responses, we measured their expression in infected mouse lungs and observed that the S8 virus induced markedly higher production of these cytokines in comparison to SDL124 (Figure 1D–F). Moreover, to assess the histopathology caused by these two viruses, mouse lungs were collected at 3 dpi for hematoxylin-eosin staining. Microscopic images of the SDL124-infected mice showed minimal signs of infection (Figure 1G), whereas S8 infection induced severe pulmonary injury characterized by hyperemia, inflammatory cells or lymphocyte infiltration in the alveolar ducts and bronchial lumen, and mild alveolar distension (Figure 1H). Collectively, these findings indicated that the S8 virus exhibits enhanced replication and elevated cytokine expression in mouse lungs compared to the SDL124 strain, which underlies its heightened virulence in mice.

### 3.2. Transcriptome Analysis of S8- and SDL124-Infected Mouse Lungs

**Figure 2:**
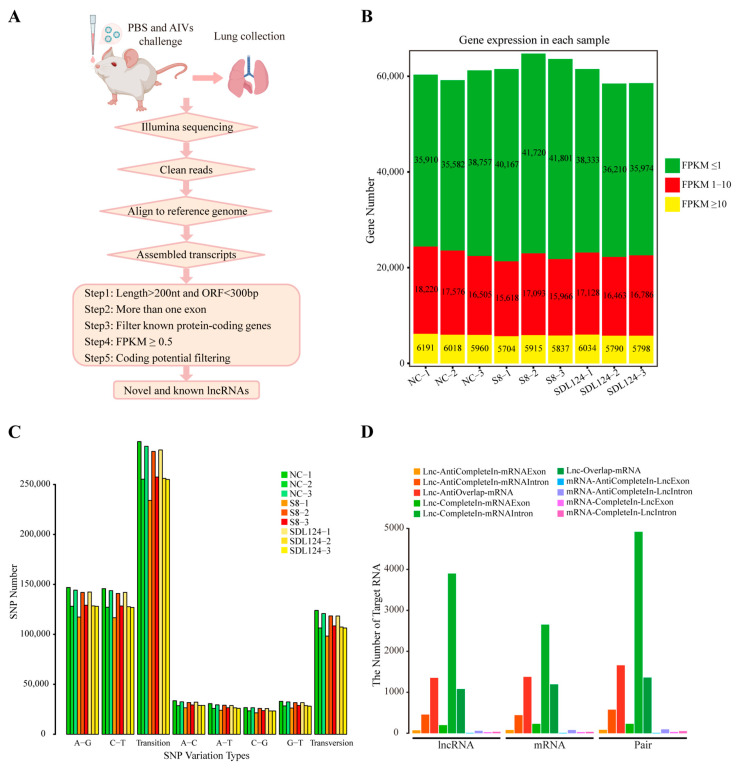
Genomic features of lncRNAs and mRNAs. (**A**) The schematic diagram of the identification pipeline for lncRNAs. (**B**) Gene expression across all samples. (**C**) SNP variation types of genes in each sample. (**D**) Classification of overlap between lncRNAs and their cis-regulated target mRNAs.

Given the significant differences in pathogenicity (viral loads, cytokine expression, and histological changes) between the two viruses (Figure 1), we conducted global transcriptome analysis of lncRNA and mRNA expression in mouse lungs at 2 dpi. Following the screening strategy for lncRNAs shown in Figure 2A, we annotated known lncRNAs and identified novel transcripts. Subsequently, systematic cluster analysis assessed inter-sample correlations, confirming the experiment reliability and sample selection validity (Figure S1). Analysis of total genes (lncRNAs and mRNAs) based on the expression level revealed that genes with FPKM ≤ 1 constituted the largest proportion in all samples, while those with FPKM ≥ 10 represented the smallest. Of note, the S8 group exhibited a significantly higher number of genes compared with both the negative control (NC; *p* = 0.048) and SDL124 (*p* = 0.022) groups (Figure 2B). Single nucleotide polymorphisms (SNPs) primarily consist of transitions and transversions. As shown in Figure 2C, transitions exceeded transversions more than two-fold in both lncRNAs and mRNAs across all samples. Additionally, we also classified overlaps between lncRNAs and their cis-regulated target mRNAs and found that Lnc-CompleteIn-mRNAIntron exhibited the highest number, while mRNA-AntiCompleteIn-LncExon showed the lowest (Figure 2D).

**Figure 3:**
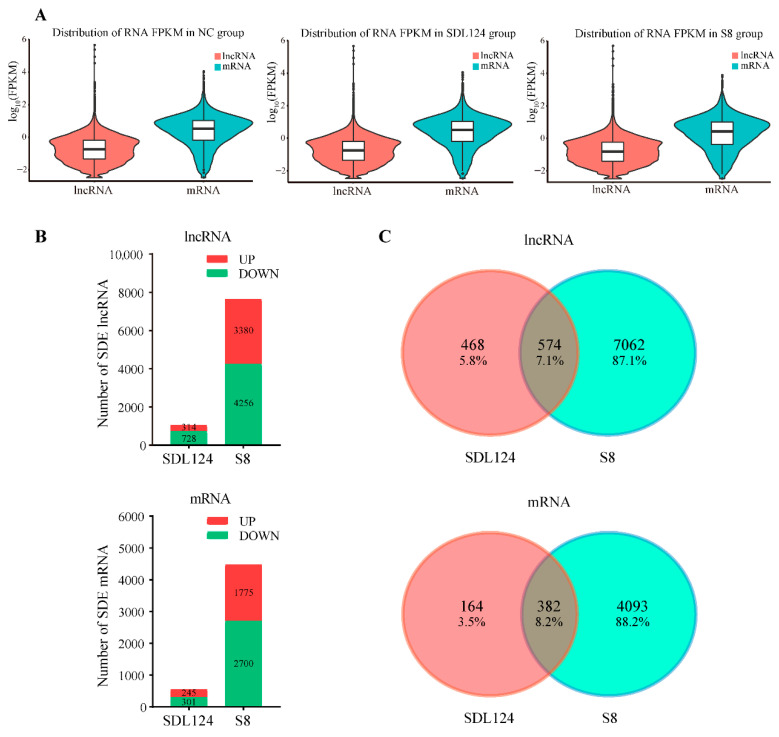
Analysis of the lncRNA and mRNA data. (**A**) FPKM density distribution of lncRNAs and mRNAs across all groups. (**B**) The number of SDE lncRNAs (**upper panel**) and mRNAs (**lower panel**) in infected groups relative to the mock group (|log_2_ fold change| ≥ 1, *p* value < 0.05 and FDR < 0.05). (**C**) Venn diagrams of SDE lncRNAs (**upper panel**) and mRNAs (**lower panel**) overlapping between the S8- and SDL124-infected groups.

Analysis of FPKM density distribution revealed distinct expression patterns between lncRNAs and mRNAs across all groups. The median expression (log_10_ FPKM) of lncRNAs in each group was below zero, whereas that of mRNAs was above zero. Moreover, the average expression of lncRNAs was consistently lower than that of mRNAs (Figure 3A). Based on the screening criteria (|log_2_ fold change| ≥ 1, *p* value < 0.05 and FDR < 0.05), we identified 7636 known SDE lncRNAs in the S8-infected group compared to the mock group, of which 3380 were up-regulated and 4256 were down-regulated. In contrast, the SDL124 group showed only 1042 known SDE lncRNAs, with 314 up-regulated and 728 down-regulated (Figure 3B, upper panel). Similarly, S8 infection altered 4475 known SDE mRNAs (1775 up-regulated and 2700 down-regulated), while only 546 SDE mRNAs (245 up-regulated and 301 down-regulated) were identified in the SDL124 group (Figure 3B, lower panel). Notably, only a small portion of SDE lncRNAs (7.1%) and mRNAs (8.2%) were shared between the S8 and SDL124 groups (Figure 3C), suggesting that genes specifically altered by S8 infection may contribute to its enhanced virulence.

### 3.3. LncRNA/mRNA Co-Expression Network

**Figure 4:**
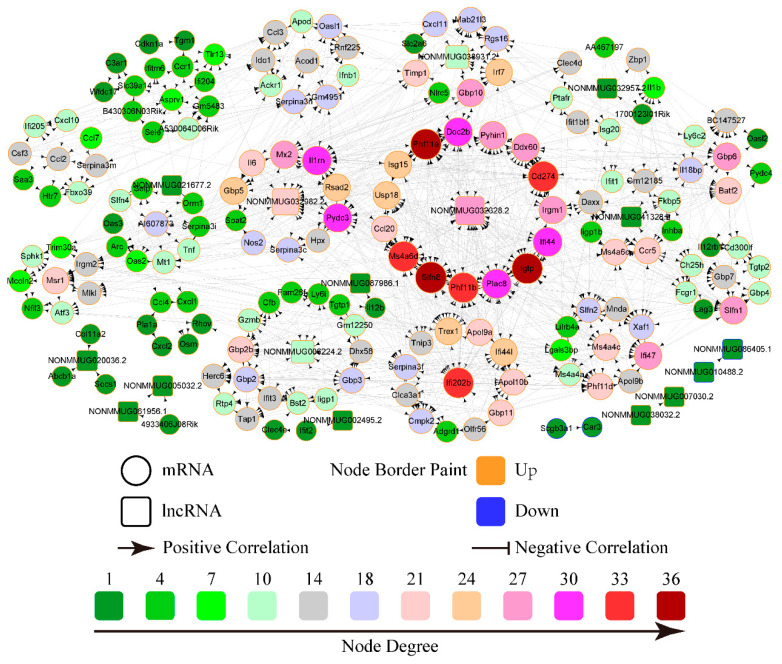
The lncRNA/mRNA co-expression network for the S8-infected group. The lncRNAs and mRNAs with |*r*| > 0.999 and FDR < 0.05 were screened to construct the network using Cytoscape v3.8.2. Each node represents a single gene (lncRNA or mRNA). An edge connecting two nodes indicates a significant correlation in their expression patterns. The degree of a node represents the number of genes directly connected to it and reflects the relative importance of the node as a potential hub gene. Node size corresponds to degree and the number (**lower panel**) corresponding to each color refers to the number of directly connected genes. Larger and darker nodes indicate higher connectivity (more genes directly linked to the target gene).

Since lncRNAs function in diverse biological processes by regulating target mRNAs, we identified SDE mRNAs co-expressed with SDE lncRNAs according to cis/trans-regulatory algorithms and constructed an lncRNA/mRNA co-expression network. In the S8-infected group, the co-expression network comprised 185 nodes (16 SDE lncRNAs and 169 SDE mRNAs) and 1062 edges (Figure 4). Several lncRNAs, including NONMMUG032982.2, NONMMUG032328.2 and NONMMUG006224.2, exhibited extensive correlations with numerous target mRNAs. Specifically, the top-ranked lncRNA, NONMMUG032328.2, targeted 27 nodes and the second-ranked lncRNA, NONMMUG032982.2, correlated with 21 nodes. Correspondingly, the top-ranked mRNA, *Slfn8*, targeted 36 nodes. Furthermore, the network revealed that NONMMUG032982.2 and NONMMUG032328.2 were highly correlated with multiple immune genes, such as *IL-6*, *CCl20*, *ISG15*, *MX2*, and *IFI44*. In contrast, the SDL124-infected group network consisted of 218 nodes (80 SDE lncRNAs and 138 SDE mRNAs) and 1088 edges (Figure S2). The top-ranked lncRNA (NONMMUG053816.1) and mRNA (*Ifi27l2a*) targeted 23 and 28 nodes, respectively. However, most co-expressed mRNAs in the SDL124 group were not related to immune responses. Overall, these results suggested that compared to the SDL124 group, lncRNAs in the S8-infected group exhibited a stronger functional association with immune responses.

### 3.4. Functional Enrichment of Target mRNAs for SDE LncRNAs

**Figure 5:**
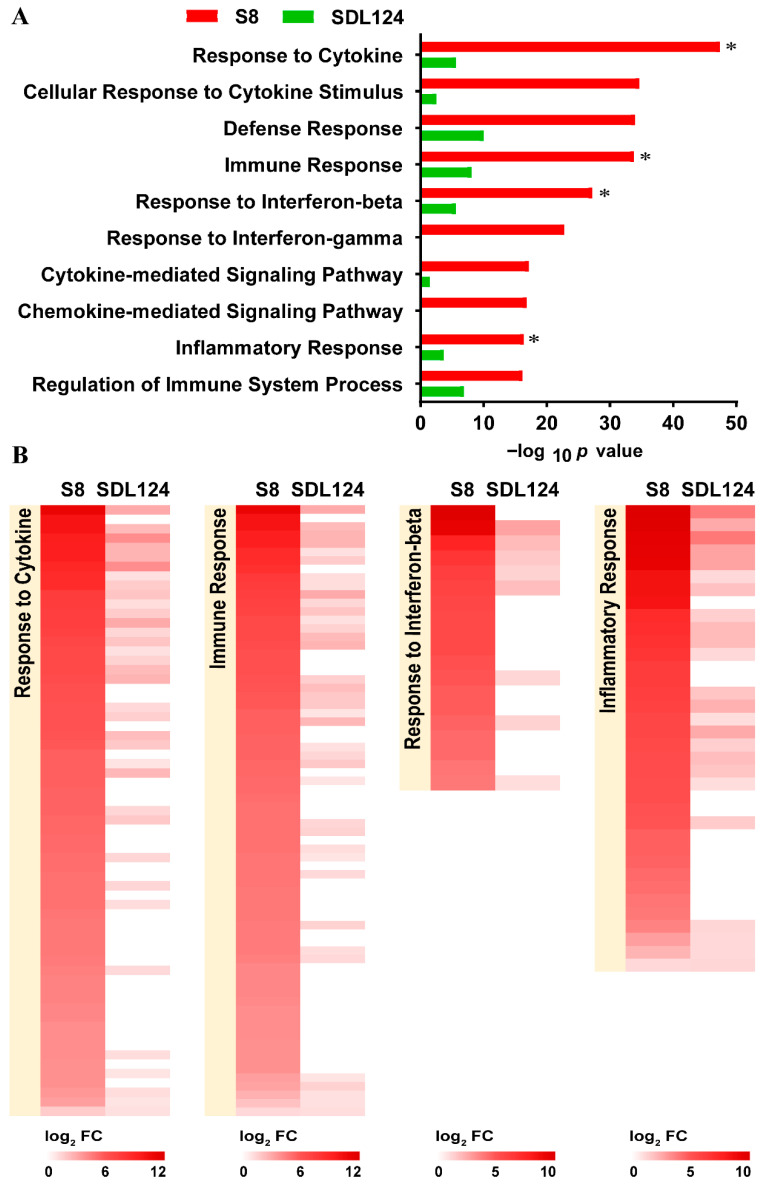
GO enrichment analysis of the lncRNA-co-expressed mRNAs induced by the S8 or SDL124 virus. (**A**) Top 10 biological processes enriched by lncRNA-co-expressed mRNAs. * represents that the −log_10_ *p* value of the S8 group is over two-fold higher than that of SDL124. (**B**) Key immunological processes significantly induced by S8 infection.

To predict the potential functions of SDE lncRNAs in S8- and SDL124-infected groups, we performed functional annotation analysis on their co-expressed mRNAs. The top 10 significantly enriched terms (according to −log_10_ *p* value) for the S8 group were summarized in Figure 5A. These results indicated that co-expressed mRNAs were primarily enriched in key immunological processes, such as response to cytokine, defense response, and response to interferon-beta. Within these terms, the number of enriched mRNAs in the S8-infected group was greater than that of SDL124. Notably, the four terms (response to cytokine, immune response, response to interferon-beta, and inflammatory response, indicated by ‘*’) in which co-expressed mRNAs were involved were above two-fold enriched in the S8-infected group compared to the SDL124 group (Figure 5A). Moreover, fold change in the upregulation of mRNAs in these terms induced by S8 infection was also higher than that induced by SDL124 (Figure 5B). Altogether, these findings showed that mRNAs co-expressed with S8-induced SDE lncRNAs were strongly correlated with immune responses, particularly inflammatory-related responses.

**Figure 6:**
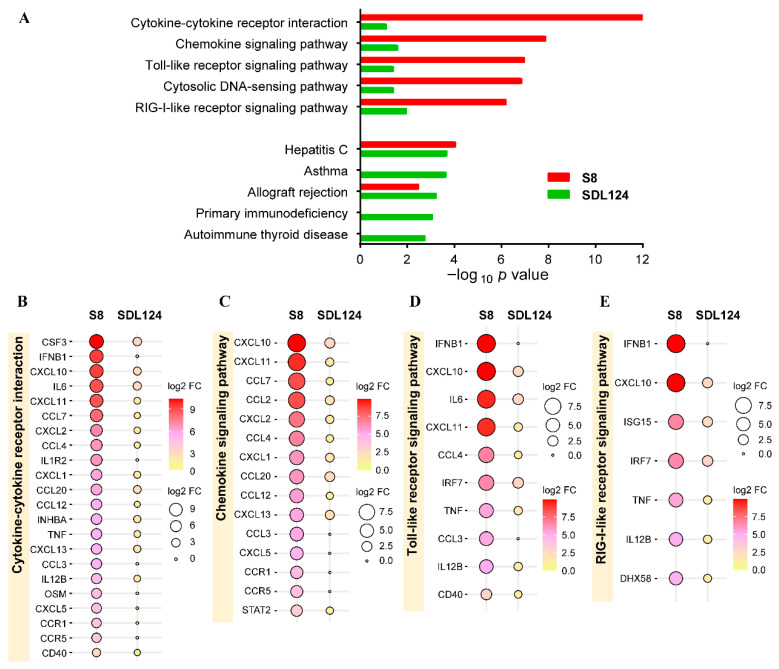
Pathway enrichment analysis of the lncRNA-co-expressed mRNAs induced by the S8 or SDL124 virus. (**A**) Top 5 enriched pathways for lncRNA-co-expressed mRNAs in the S8 and SDL124 groups. (**B**) Expression profiles of lncRNA-co-expressed mRNAs enriched in the top-ranked pathway for the S8 group. (**C**) Expression profiles of lncRNA-co-expressed mRNAs enriched in the second-ranked pathway for the S8 group. (**D**) Expression profiles of lncRNA-co-expressed mRNAs enriched in the third-ranked pathway for the S8 group. (**E**) Expression profiles of lncRNA-co-expressed mRNAs enriched in the fifth-ranked pathway for the S8 group.

We further performed KEGG enrichment analysis to identify pathways enriched for mRNAs co-expressed with lncRNAs. As shown in Figure 6A, the top five enriched pathways in the S8 group comprised cytokine-cytokine receptor interaction, chemokine signaling pathway, Toll-like receptor signaling pathway, cytosolic DNA-sensing pathway, and RIG-I-like receptor signaling pathway. Therefore, based on these results, we surmised that lncRNA-co-expressed mRNAs in the S8 group were predominantly associated with innate immune signaling pathways. In contrast, the top 5 pathways enriched in the SDL124 group included hepatitis C, asthma, allograft rejection, primary immunodeficiency, and autoimmune thyroid disease (Figure 6A). Thus, the analysis results for the top five enriched pathways showed that there was no obvious overlap between the two groups. In addition, compared to the SDL124 group, the co-expressed mRNAs associated with four specific immune-related pathways in the S8 group were more markedly up-regulated (Figure 6B–E). Taken together, these results demonstrated that lncRNA-co-expressed mRNAs induced by S8 were highly associated with inflammatory-related pathways, which was consistent with the GO enrichment results.

### 3.5. Validation of SDE LncRNAs and mRNAs by RT-qPCR

**Figure 7:**
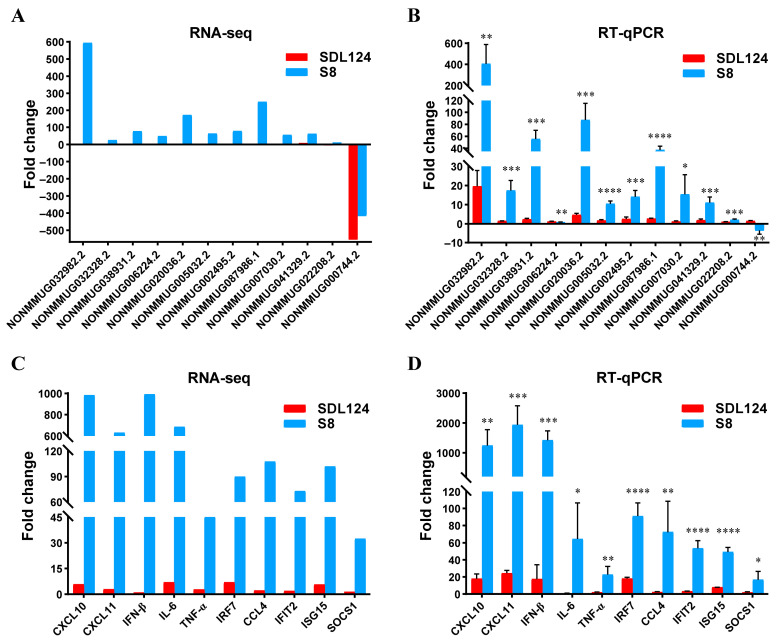
Validation of the RNA-seq data by RT-qPCR. (**A**) RNA-seq results of selected SDE lncRNAs were used as a control. (**B**) Expression of SDE lncRNAs in S8- or SDL124-infected lungs at 2 dpi was validated by RT-qPCR. (**C**) RNA-seq results of selected mRNAs were used for comparison. (**D**) Expression of the selected mRNAs in S8- or SDL124-infected lungs at 2 dpi was validated by RT-qPCR. Data shown are the mean ± SD from three biological replicates. * represents comparison with the SDL124 group. * (*p* < 0.05), ** (*p* < 0.01), *** (*p* < 0.001), and **** (*p* < 0.0001) are considered statistically significant.

To validate the reliability of RNA-seq data derived from S8- and SDL124-infected mouse lungs, 12 SDE lncRNAs and 10 mRNAs co-expressed with SDE lncRNAs were selected for verification by RT-qPCR. The RT-qPCR results showed that lncRNAs displayed similar expression trends to RNA-seq results (Figure 7A,B). Similarly, the expression of 10 selected mRNAs detected by RT-qPCR was also highly consistent with those obtained by RNA-seq (Figure 7C,D). Furthermore, RT-qPCR analysis revealed significantly higher expression of all selected lncRNAs and mRNAs in the S8 group relative to the SDL124 group, except for NONMMUG006224.2 and NONMMUG000744.2 (Figure 7B,D). Collectively, these findings confirmed the accuracy and high reproducibility of the sequencing data.

### 3.6. Potential Roles of NONMMUG032982.2 and NONMMUG032328.2 During H7N9 AIV Infection

**Figure 8:**
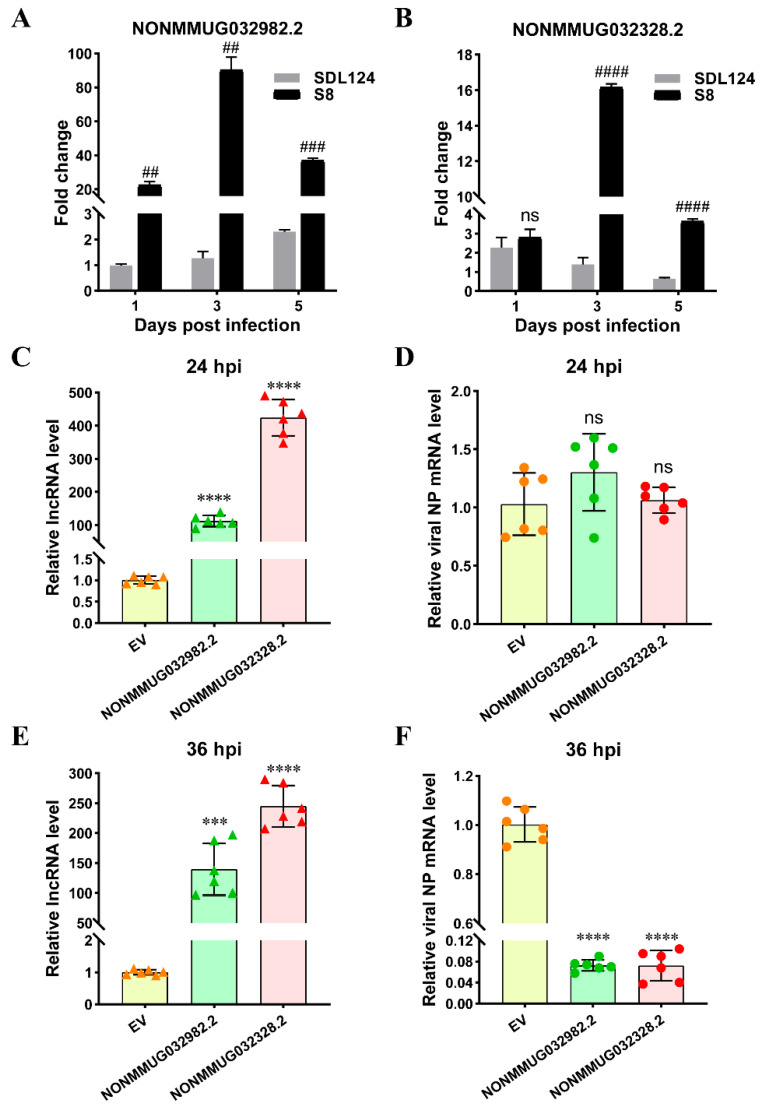
Potential roles of NONMMUG032982.2 and NONMMUG032328.2 during H7N9 AIV infection. (**A**,**B**) Groups of nine mice were intranasally inoculated with 10^5^ EID_50_ in 50 μL PBS of S8 or SDL124 virus. (**A**) The expression pattern of NONMMUG032982.2 was detected in the lungs at 1, 3 and 5 dpi. (**B**) The expression pattern of NONMMUG032328.2 was detected in the lungs at 1, 3 and 5 dpi. (**C**,**F**) L929 cells were transfected with lncRNA plasmids (NONMMUG032982.2 or NONMMUG032328.2) or EV for 24 h, followed by infection with the S8 virus at a MOI of 0.01. (**C**) The overexpression of both lncRNAs was verified by RT-qPCR at 24 hpi. (**D**) Expression of viral NP mRNA was detected by RT-qPCR at 24 hpi. (**E**) The overexpression of both lncRNAs was confirmed by RT-qPCR at 36 hpi. (**F**) Expression of viral NP mRNA was detected by RT-qPCR at 36 hpi. Data represent the mean ± SD of the results. Triangles and circles represent the values of technical replicates. # indicates comparison with the SDL124 group. * indicates comparison with the mock group (EV). ## (*p* < 0.01), *** and ### (*p* < 0.001), **** and #### (*p* < 0.0001) are considered statistically significant, and ns indicates non-significance.

Our previous studies indicated that lncRNAs NONMMUG032328.2 and NONMMUG032982.2 targeted 27 and 21 nodes in the co-expression network, respectively, and exhibited high correlation with multiple immune genes (Figure 4), suggesting their potential involvement in AIV replication. Therefore, we collected lung samples from mice infected with S8 or SDL124 viruses at 1, 3 and 5 dpi and determined the expression patterns of these two lncRNAs in mouse lungs. As shown in Figure 8A,B, in the S8-infected group, both lncRNAs displayed significantly higher expression levels compared to the SDL124-infected group at most time points. Moreover, both lncRNAs in S8-infected mice exhibited a common expression trend, with expression first increasing at 3 dpi, subsequently decreasing at 5 dpi, and peaking at 3 dpi, which was strongly consistent with the viral titer results (Figure 1C). Therefore, we further investigated the potential roles of NONMMUG032982.2 and NONMMUG032328.2 during S8 virus infection in vitro. We first confirmed the successful overexpression of both lncRNAs in L929 cells (Figure 8C,E), and then we found that they had no significant effect on NP mRNA levels of S8 virus at 24 hpi (Figure 8D). However, by 36 hpi, forced expression of these two lncRNAs significantly inhibited S8 virus replication (Figure 8F). Altogether, these data provided the first preliminary evidence that NONMMUG032982.2 and NONMMUG032328.2 exerted antiviral effects against AIV.

### 3.7. Bioinformatics Analysis of Both Functional LncRNAs

**Figure 9:**
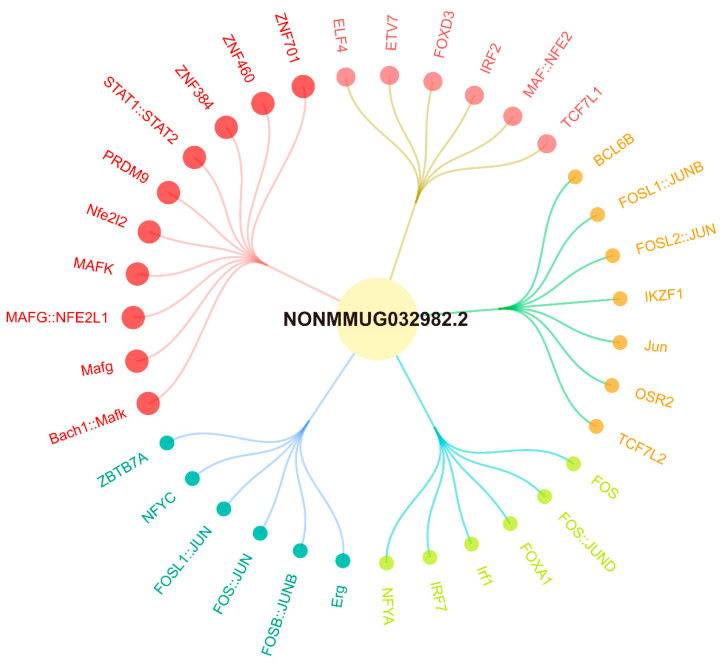
Prediction of potential TFs for NONMMUG032982.2. The larger the shape and the darker the color indicate that the indicated TF harbors a lower *p* value matching the lncRNA promoter site. Normally, a lower *p* value indicates a higher reliability.

**Figure 10:**
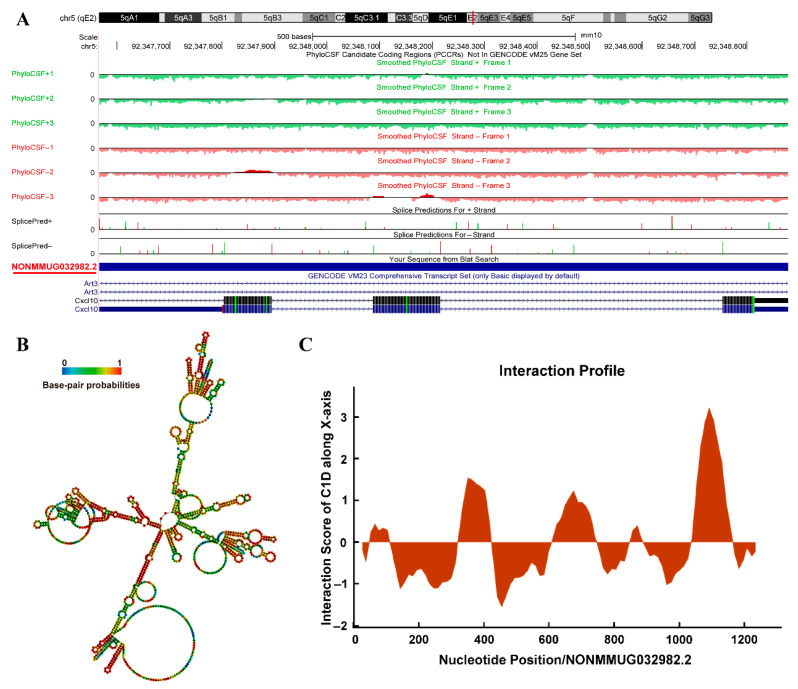
Bioinformatics analysis of NONMMUG032982.2. (**A**) The genomic location and coding potential of NONMMUG032982.2 in the mouse genome were characterized. (**B**) Secondary structure prediction of NONMMUG032982.2. Shown is an optimal minimum free energy structure (MFE = −399.20 kcal/mol). (**C**) C1D exhibited high binding affinity for NONMMUG032982.2.

Our results demonstrate that both lncRNAs play a role in suppressing S8 virus replication. To further explore their potential biological functions, we conducted systematic bioinformatics analyses. By integrating the UCSC Genome Browser and JASPAR database, we predicted that 35 TFs would bind to the promoter region of NONMMUG032982.2, including MAFG (maf bZIP transcription factor G), STAT1::STAT2 (heterodimer), Nfe2l2 (encoding NRF2), and ZNF (zinc finger) family members (Figure 9). For NONMMUG032328.2, 34 potential TF-binding partners were predicted, such as PRDM9 (PR/SET domain containing protein 9), KLF15 (krüppel-like factor 15), RREB1 (ras-responsive element binding protein 1), and ZNF family proteins (Figure S3). Using the UCSC Genome Browser, we annotated the genomic locations of these lncRNAs and found that NONMMUG032982.2 is located on chromosome 5qE2 and NONMMUG032328.2 is located on chromosome 5qB3 (Figure 10A and Figure S4A). PhyloCSF tracks analysis assessed their coding potential, confirming that neither lncRNA encodes a protein (Figure 10A and Figure S4A). Secondary structure predictions via the RNAfold web server indicated that both lncRNAs adopted highly folded conformations featuring multiple hairpin loops (Figure 10B and Figure S4B). Furthermore, potential interacting proteins for both lncRNAs were predicted using catRAPID. For NONMMUG032982.2, several proteins showed strong interaction potential, including C1D, SARNP (SAP domain-containing ribonucleoprotein), PABP2 (polyadenylate-binding protein 2), and NIP7 (nucleolar pre-rRNA processing protein). Notably, C1D exhibited high binding affinity specifically for the 3′-terminal region of this lncRNA (Figure 10C; Table 1). Similarly, NONMMUG032328.2 was predicted to interact tightly with proteins such as KHDR1 (KH domain-containing, RNA-binding, signal transduction-associated protein 1), G3BP1 (ras GTPase-activated protein-binding protein 1) and PNO1 (partner of NOB1 homolog), and KHDR1 showed strong binding to its 5′-terminal region (Figure S4C; Table S2).

## 4. Discussion

Substantial evidence indicates that lncRNAs have become a major focus in non-coding RNA research [11,16]. Despite low conservation, they play diverse and pivotal roles in various biological processes [38]. Currently, the functions of lncRNAs in numerous viral infections have been increasingly revealed [39,40]. Nevertheless, their specific regulatory roles during AIV infection remain incompletely understood. In this study, we analyzed lncRNA and mRNA expression profiles in mouse lungs infected either with the parental SDL124 strain or the mouse-adapted S8 variant. A total of 7636 known SDE lncRNAs were identified in the S8-infected group, whereas the SDL124 group showed only 1042 (Figure 3B). Furthermore, lncRNA-co-expressed mRNAs induced by S8 infection displayed strong enrichment for inflammatory responses (Figure 4, Figure 5 and Figure 6). Most significantly, for the first time, we preliminarily confirmed that NONMMUG032982.2 and NONMMUG032328.2 possessed antiviral activity against AIV (Figure 8F).

SNPs primarily consist of transitions and transversions. These variations can alter lncRNA structure, thereby affecting its function [41]. In the present study, we observed that transitions occurred more than twice as frequently as transversions across all samples (Figure 2C). Within the animal kingdom, mutations from A to G may be caused by RNA editing mediated by the ADAR (adenosine deaminase) RNA editing enzyme. If they occur in the protein-coding region of a gene, they may change the coding of amino acids. However, in the vast majority of metazoans from sponges to humans, A-to-G mutations primarily occur in non-coding RNAs transcribed from genomic repeat sequences, particularly young repeats [42,43]. Crucially, A-to-G editing plays vital roles in diverse biological processes, including development, growth, and environmental adaptation in animals. Dysregulation of this editing mechanism is implicated in human diseases such as immune disorders, neurological disorders, and cancer [42,44]. Moreover, we analyzed overlaps between lncRNAs and their cis-acting target mRNAs and found that Lnc-CompleteIn-mRNAIntron displayed the highest number (Figure 2D), indicating that lncRNAs are mainly located in the intron region of mRNAs on the same chain.

Inflammation is a physiological response to viral infection [7]. The results of the co-expression network analysis revealed that lncRNAs in the S8-infected group exhibited a strong functional association with immune responses (Figure 4). Functional annotation analysis demonstrated that mRNAs co-expressed with S8-induced SDE lncRNAs were significantly correlated with inflammatory responses (Figure 5). Pathway enrichment analysis further indicated that these co-expressed mRNAs in the S8 group were significantly enriched for inflammatory-related pathways (Figure 6). Additionally, co-expressed mRNAs in the S8 group displayed higher expression levels than those in the SDL124 group in the enrichment analysis. Based on these findings, we suspect that S8-induced SDE lncRNAs may contribute to excessive inflammatory responses. In a previous study, we found that inflammatory cell infiltration induced by the S8 virus enhanced its virulence in mice [3]. Here, we also observed that the S8 virus induced markedly higher cytokine production (Figure 1D–F). Therefore, the potential roles of lncRNAs in S8 virus-induced mouse death warrant further investigation.

Thus far, numerous studies have established critical roles for lncRNAs in the regulation of AIV infection [29,30,32,33,45,46,47]. For instance, deletion of lncRNA *USP30-AS1* enhances viral protein synthesis, viral growth, and pro-inflammatory responses in IAV-infected cells, suggesting its role as a key modulator of immune responses in IAV infection [29]. However, contradictory findings also exist, with another study demonstrating that *USP30-AS1* directly binds PHB1, modulates its protein stability, impedes nuclear import of IRF3, and consequently promotes IAV replication [32]. Mouse lnc-Cxcl2 binds the *Cxcl2* promotor and inhibits its transcription in *cis*, thereby restraining neutrophil-mediated lung inflammation in IAV infection [45]. In addition, lncRNA GBP1P1 acts as a decoy to compete with viral mRNAs for DHX9 binding, and subsequently restricts IAV replication [30], while lncRNA GAPLINC functions as a critical regulator involved in the promotion of IAV replication by ATG7 [33]. However, Lnc-ALOX12 promotes IAV infection by binding to IAV RNA polymerase subunit PB2 and stabilizing PB2-importin-α/β interaction, thereby facilitating PB2 nuclear import and efficient viral RNA synthesis [46]. In our study, both NONMMUG032982.2 and NONMMUG032328.2 exhibited expression patterns consistent with the S8 virus titer kinetics in infected mouse lungs (Figure 8A,B). Thus, we further investigated their roles in S8 virus infection and surprisingly found that both lncRNAs inhibited viral replication. However, further studies are needed to elucidate the precise underlying mechanisms by which these lncRNAs regulate AIV replication.

TFs play a pivotal role in regulating gene expression by interacting with DNA regulatory elements known as promoters [48]. Herein, we predicted 35 potential TF-binding partners for NONMMUG032982.2, including MAFG, STAT1::STAT2, Nfe2l2 (encoding NRF2), and ZNF family members (Figure 9). For instance, increased MAFG signaling during diet-induced obesity (DIO) suppresses *LinclRS2* expression. Intriguingly, silencing MAFG de-represses *LinclRS2* and improves glucose metabolism, ultimately identifying a MAFG-lncRNA axis controlling hepatic glucose metabolism [49]. ZNF460 plays a role in various cancer progressions promoted by lncRNAs [50,51,52]. It can bind the LncSNHG14 promoter to induce its transcription, facilitating gastric cancer (GC) progression [50]. Moreover, ZNF460 enhances circRPPH1 expression to promote triple-negative breast cancer (TNBC) progression [51] and positively regulates LINC00857 expression to facilitate pancreatic adenocarcinoma (PAAD) cell progression [52]. Transcriptional enhancement of lncRNA CARMN through the binding of NRF2 to its promoter region prevents abdominal aortic aneurysm (AAA) formation [53]. However, the mechanisms by which TFs regulate lncRNA expression, as well as their role in lncRNA-mediated anti-AIV effects, remain to be further investigated.

lncRNAs can regulate viral replication and host immune responses by interacting with host proteins [30,32,46,54]. In this study, several proteins displayed strong interaction potential with NONMMUG032982.2, such as C1D, SARNP, PABP2 and NIP7 (Figure 10C; Table 1). The RNA-binding protein, C1D, associates with human exosome subunit PM/Scl-100 and participates in pre-rRNA processing [55]. C1D also interacts with condensin SMC hinge and supports the DNA repair function of condensin [56]. SARNP, an mRNA nuclear export protein, can normalize neuronal activity and autistic behaviors in UBE3A-overexpressing mice by promoting nuclear mRNA export and protein translation of a key AMPAR subunit [57]. Furthermore, the structural basis for the high-order complex of SARNP and DDX39B facilitates mRNP assembly and export [58]. PABP2 binds to lncRNA ZFAS1 to stabilize SREBP1 mRNA, thereby increasing the expression of SREBP1 and its target genes SCD1 and FASN, thus promoting lipid accumulation in colorectal cancer (CRC) [59]. NIP7 plays a role in pre-rRNA maturation in human cells [60]. However, unfortunately, these studies did not elucidate the potential roles of these specific proteins in AIV infection and host immune responses, which will be the focus of our future research.

In summary, we identified the functional SDE lncRNAs and performed enrichment analysis of lncRNA-co-expressed mRNAs in the avirulent or virulent H7N9 virus-infected mouse lung. Furthermore, we reported for the first time that NONMMUG032982.2 and NONMMUG032328.2 exhibited antiviral activity against AIV. Through bioinformatic analysis, we have also gained deeper insights into these two functional lncRNAs. These findings enhance our understanding of the diverse roles of lncRNAs in viral infections, while also providing a foundation for further research into the mechanisms of lncRNAs during AIV infection.

## Figures and Tables

**Table 1:** Top proteins that might interact with NONMMUG032982.2.

Protein Name	Amino Acids	Interaction Strength (%)	Discriminative Power (%)
C1D	141	100	94
THOC4	255	100	76
U2AF1	239	100	76
SARNP	210	100	92
PABP2	302	99	76
NIP7	180	99	92
PAI2B	136	99	92
NH2L1	128	99	92

## Data Availability

The RNA-sequencing data from this study have been deposited in NCBI GEO under accession number GSE304574 (https://www.ncbi.nlm.nih.gov/geo/query/acc.cgi?acc=GSE304574, accessed on 10 August 2025). Raw numerical data and statistical calculations for plotting graphs generated in this study are deposited in Figshare (DOI: https://doi.org/10.6084/m9.figshare.29634419.v2, accessed on 10 August 2025). The genomic sequences of the S8 and SDL124 viruses are available in GenBank under the accession numbers MW397099 to MW397114.

## Supplementary Materials

The following supporting information can be downloaded at: https://www.mdpi.com/article/10.3390/v18030353/s1, Figure S1: Heatmap of correlation between samples. Figure S2: The lncRNA/mRNA co-expression network for the SDL124-infected group. Figure S3: Prediction of the potential TFs for NONMMUG032328.2. Figure S4: Bioinformatics analysis of NONMMUG032328.2. Table S1: RT-qPCR primers used for the identification of lncRNAs and mRNAs in this study. Table S2: Top protein that might interact with NONMMUG032328.2.
