# Supplementary material for: Systematic Identification of the Functional lncRNAs During H7N9 Avian Influenza Virus Infection in Mice
Source: Viruses. 2026 Mar 13;18(3):353. doi: 10.3390/v18030353 (PMC13030536; doi:10.3390/v18030353)
Supplement: Supplementary file 1 [file viruses-18-00353-s001.zip › Figure S1.pdf]

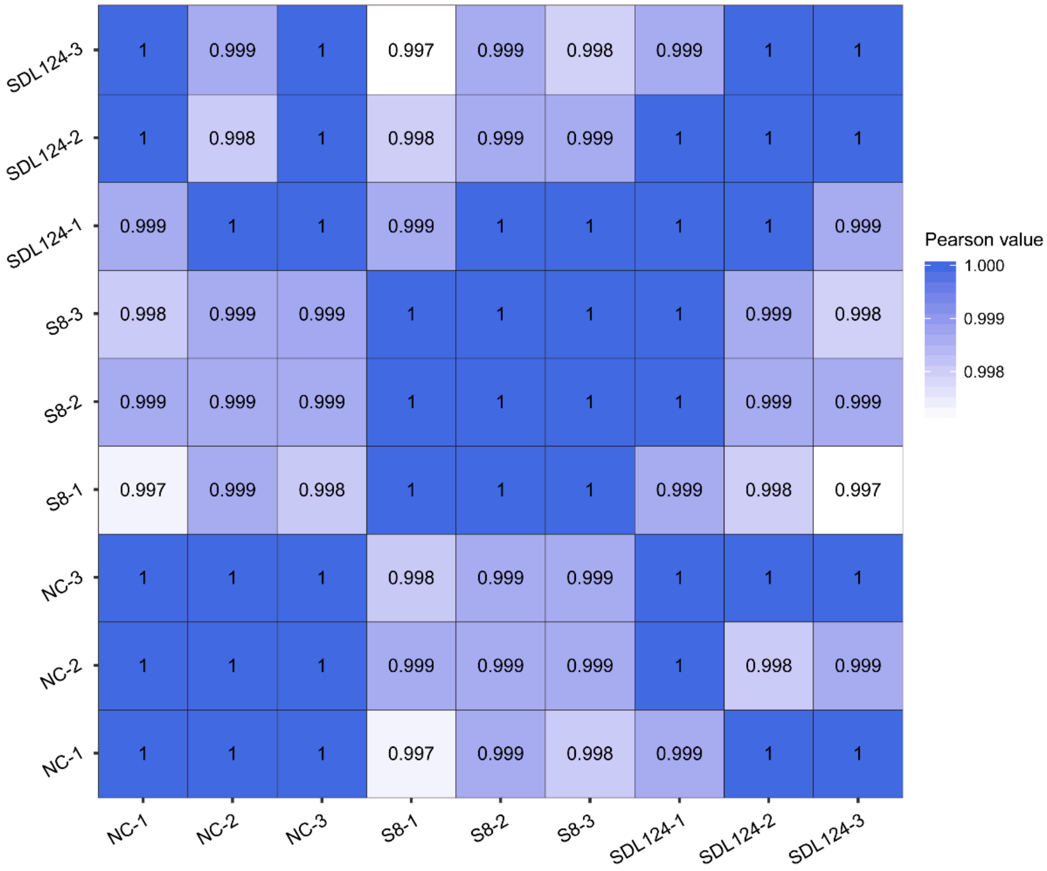

Figure S1. Heatmap of correlation between samples. The correlation between samples was calculated based on FPKM values of genes, and an inter-sample correlation heatmap was generated using *R*.
