# Supplementary material for: Systematic Identification of the Functional lncRNAs During H7N9 Avian Influenza Virus Infection in Mice
Source: Viruses. 2026 Mar 13;18(3):353. doi: 10.3390/v18030353 (PMC13030536; doi:10.3390/v18030353)
Supplement: Supplementary file 1 [file viruses-18-00353-s001.zip › Figure S2.pdf]

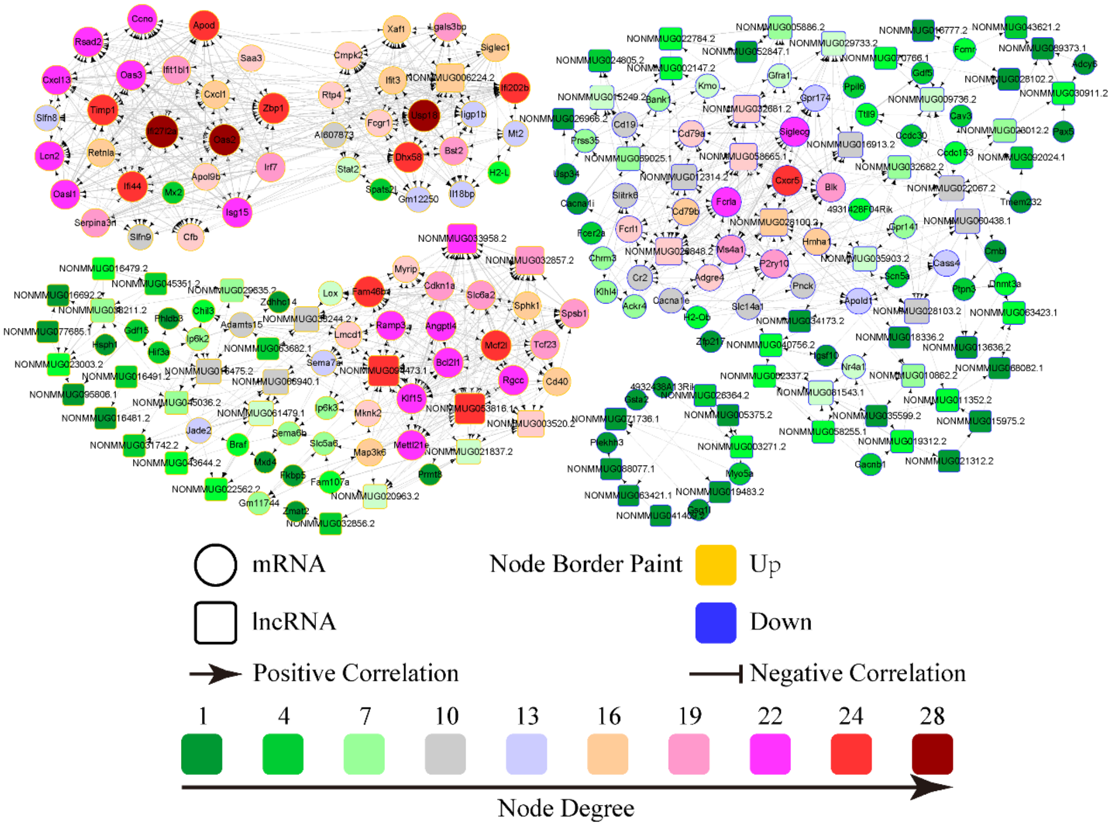

Figure S2. The lncRNA/mRNA co-expression network for the SDL124-infected group. The lncRNAs and mRNAs with $|r| > 0.98$ and FDR < 0.05 were screened to construct the network using Cytoscape v3.8.2. Each node represents a single gene (lncRNA and mRNA). An edge connecting two nodes indicates a significant correlation in their expression patterns. The degree of a node represents the number of genes directly connected to it and reflects the relative importance of the node as a potential hub gene. Node size corresponds to degree and the number (lower panel) corresponding to each color refers to the number of directly connected genes. Larger and darker nodes indicate higher connectivity (more genes directly linked to the target gene).
