# Supplementary material for: Systematic Identification of the Functional lncRNAs During H7N9 Avian Influenza Virus Infection in Mice
Source: Viruses. 2026 Mar 13;18(3):353. doi: 10.3390/v18030353 (PMC13030536; doi:10.3390/v18030353)
Supplement: Supplementary file 1 [file viruses-18-00353-s001.zip › Figure S3.pdf]

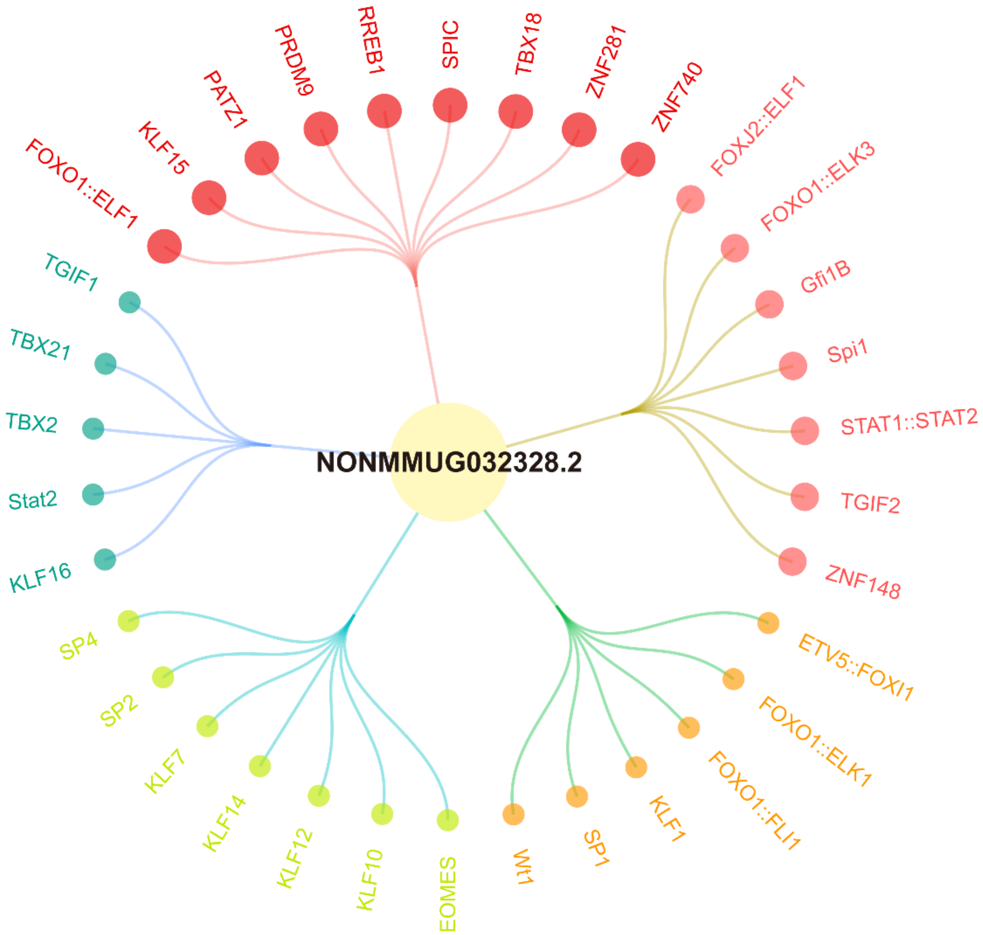

Figure S3. Prediction of the potential TFs for NONMMUG032328.2. The larger the shape and the darker the color represent that the indicated TF harbors a lower *p* value matching the lncRNA promoter site. Normally, a lower *p* value indicates a higher reliability.
