# Supplementary material for: Systematic Identification of the Functional lncRNAs During H7N9 Avian Influenza Virus Infection in Mice
Source: Viruses. 2026 Mar 13;18(3):353. doi: 10.3390/v18030353 (PMC13030536; doi:10.3390/v18030353)
Supplement: Supplementary file 1 [file viruses-18-00353-s001.zip › Figure S4.pdf]

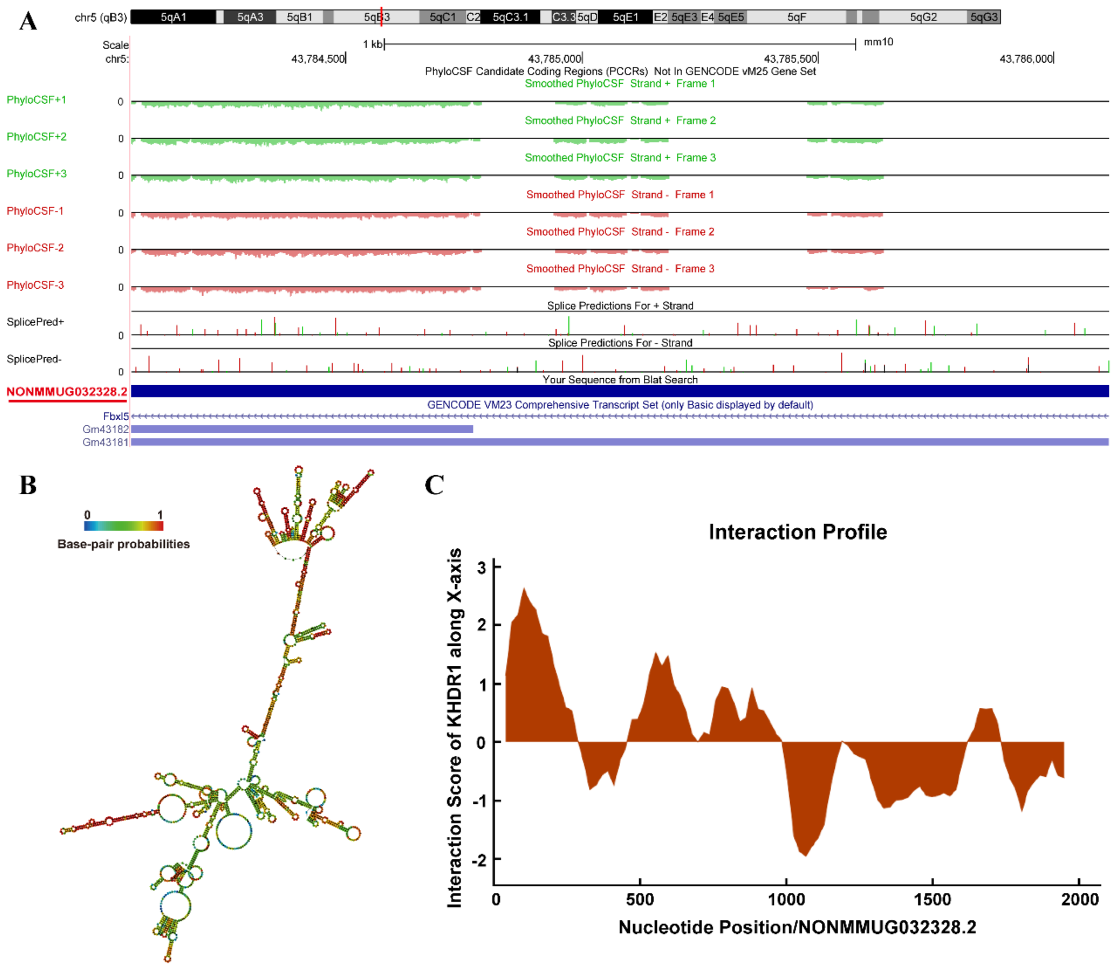

Figure S4. Bioinformatics analysis of NONMMUG032328.2. (A) The genomic location and coding potential of NONMMUG032328.2 in the mouse genome were characterized. (B) Secondary structure prediction of NONMMUG032328.2. Shown is an optimal minimum free energy structure (MFE= -611.70 kcal/mol) (C) KHDR1 showed strong binding to NONMMUG032328.2.
