# Supplementary material for: Systematic Identification of the Functional lncRNAs During H7N9 Avian Influenza Virus Infection in Mice
Source: Viruses. 2026 Mar 13;18(3):353. doi: 10.3390/v18030353 (PMC13030536; doi:10.3390/v18030353)
Supplement: Supplementary file 1 [file viruses-18-00353-s001.zip › Table S1.pdf]

Table S1. qPCR primers used for the identification of lncRNAs and mRNAs in this study.

Gene/lncRNA	Primers sequence (5′ to 3′)[a]	Gene/mRNA	Primers sequence (5′ to 3′)[a]
NONMMUG032982.2	F: *ATCCCTTCAGCTTGCAGAGT*	β-actin	F: *CATCCGTAAAGACCTCTATGCCAAC*
	R: *TTTCGCCCTTTCTCCTGACT*		R: *ATGGAGCCACCGATCCACA*
NONMMUG032328.2	F: *GCCCTTCTTGTCGATGTGAC*	CXCL10	F: *CCAAGTGCTGCCGTCATTTTC*
	R: *TACCTTGCCCTCACTTCCTG*		R: *GGCTCGCAGGGATGATTTCAA*
NONMMUG020036.2	F: *AGCCTGTAGCAGTGAAGGTT*	CXCL11	F: *TCCAAAGCCCAGGCAGAGA*
	R: *GGGCATACTCCTGAAAGCCT*		R: *AGGGCTATGGCTGTGACCTTC*
NONMMUG002495.2	F: *GCCTGGCTATTTCAGCACTC*	IFN-β	F: *ATCAACCTCACCTACAGGGC*
	R: *GGCTTGAGTAGGTAGGCAGA*		R: *ATCTCTTGGATGGCAAAGGCA*
NONMMUG087986.1	F: *AGCAACAGTGCCAAGAAGTG*	IL-6	F: *TGAGATCTACTCGGCAAACCTAGTG*
	R: *GCTGCAGAGATCTGAGGACT*		R: *CTTCGTAGAGAACAACATAAGTCAGATACC*
NONMMUG038931.2	F: *CAGGCATCACGCAAGCTAAT*	IRF7	F: *CTGGAAGACCAACTTCCGCT*
	R: *CCTTCAAAGCCAACCTGGAC*		R: *TACTGCAGAACCTGTGTGGG*
NONMMUG005032.2	F: *TTGCAGTCAGGACAACTTGC*	ISG15	F: *GGTGTCCGTGACTAACTCCAT*
	R: *GCTGGCGAGATAGCTCAATG*		R: *TGGAAAGGGTAAGACCGTCCT*
NONMMUG007030.2	F: *ACATTGGGATGAGGGCAAGA*	CCL4	F: *CTCTCTCTCCTCTTGCTCGTG*
	R: *AAGCCATTCCTGACTCCACA*		R: *CTGCCGGGAGGTGTAAGAGA*
NONMMUG041329.2	F: *CTTTCCTCCCGCTCCTGTTA*	TNF-α	F: *GACTAGCCAGGAGGGAGAACAGA*
	R: *GAGCGCCCTCTGAACTCTAT*		R: *CCTGGTTGGCTGCTTGCTT*
NONMMUG000744.2	F: *GGTTCCTGGCCCATCTTAGT*	IFIT2	F: *GAGGACAGGGTGAGTCAGAAGGG*
	R: *AGACTTACCGTGTCAGAAGCA*		R: *GGAGGGCAGAGTAGGGAGACATC*
NONMMUG006224.2	F: *AGCTGTCTTCAGACACACCA*	SOCS1	F: *CGAGACCTTCGACTGCCTTT*
	R: *TGGCTCAATGGGTAAGAGCA*		R: *AGTCACGGAGTACCGGGTTA*
NONMMUG022208.2	F: *CTTAGTCTCGTGTCCGCTCT*	NP	F: *AGAGACGGAAAATGGGTGAGAGAGC*
	R: *TGACCCTTTCTGACCTCCAC*		R: *GGATCCATTCCAGTACGCACGAGAG*

[a] F, forward primer; R, reverse primer.
