# Supplementary material for: Systematic Identification of the Functional lncRNAs During H7N9 Avian Influenza Virus Infection in Mice
Source: Viruses. 2026 Mar 13;18(3):353. doi: 10.3390/v18030353 (PMC13030536; doi:10.3390/v18030353)
Supplement: Supplementary file 1 [file viruses-18-00353-s001.zip › Table S2.pdf]

Table S2. Top protein that might interact with NONMMUG032328.2.

Protein name	Amino acids	Interaction Strength (%)	Discriminative Power (%)
KHDR1	443	100	62
SBDS	250	100	58
G3BP1	465	99	59
G3BP2	482	99	59
IF4H	248	99	56
TRMB	268	99	56
PNO1	248	99	58
RT05	432	99	57
